# Comprehensive assessment of the true sepsis burden using electronic health record screening augmented by natural language processing

**DOI:** 10.1186/cc13434

**Published:** 2014-03-17

**Authors:** R Arnold, J Isserman, S Smola, E Jackson

**Affiliations:** 1 Christiana Care Health System, Newark, DE, USA

## Introduction

Interventional trials for sepsis have not shown an improvement in patient outcomes, often due to the lack of a diagnostic gold standard resulting in large heterogeneity of the patients enrolled. Electronic health record (EHR) screening tools have been applied to the sepsis population but limited to vital sign and laboratory data to identify target patients. Our objective was to describe an investigational database created through the application of a new EHR screening tool that applies natural language processing (NLP) analysis to clinical documentation to augment the identification of infection.

## Methods

We acquired data from the Clinical Vigilance for Sepsis EHR screen on consecutive patients from two hospital systems over 12 months at a 300-bed community hospital and 24 months from a 500- bed academic tertiary care center. A physician order for intravenous antibiotics was used as a surrogate for suspected infection, removing patients receiving a single dose of antibiotics without subsequent administration. Each patient's in-hospital course was tracked from arrival to final disposition, identifying vital signs, laboratory values, radiological results, and interventions as they occurred. Patients were followed for the primary outcome of mortality, with secondary outcomes of transfer to the ICU, vasopressor initiation and mechanical ventilation.

## Results

The EHR screen identified 216,550 patients over a total of 36 months from the two hospitals. A total of 37,160 (17%) patients were treated with i.v. antibiotics. Sepsis patients experienced a 3% (1,186/37,160) mortality rate relative to 0.5% (448/216,550) in patients without infection at any time. Sepsis at any time represented 73% (1,186/1,634) of all in-hospital deaths. ICU transfer occurred in 18% (6,865/37,160) of patients, with septic shock (vasopressor requirement) occurring in 10% (3,837), and 13% (5,072) requiring mechanical ventilation.

## Conclusion

Application of this novel EHR screening tool to identify sepsis patients utilizes NLP applied to clinical documentation, providing greater clinical context than laboratory and vital sign screening alone. This database represents the entire sepsis acuity spectrum, allowing for a more granular description of the infectious process as well as subgroups with adequate sample size. The dataset collected as a patient-centered time series will enable future studies to focus on the trajectory of clinical deterioration and shock before its occurrence.

